# A Randomized Controlled Trial on the Influence of Prenatal Counseling on the Attitudes and Preferences Toward Invasive Prenatal Testing Among Women in Their First Trimester of Pregnancy (INVASIVE)

**DOI:** 10.3389/fgene.2020.561283

**Published:** 2020-11-09

**Authors:** Fernanda Paz y Miño, Raigam Jafet Martinez-Portilla, Montse Pauta, Antoni Borrell

**Affiliations:** ^1^Fetal Medicine Research Center, BCNatal – Barcelona Center for Maternal-Fetal and Neonatal Medicine (Hospital Clínic and Hospital Sant Joan de Deu), Institut Clínic de Ginecologia, Obstetricia i Neonatologia, Universitat de Barcelona, Barcelona, Spain; ^2^Prenatal Diagnosis Unit, Hospital Clínic de Barcelona, Universitat de Barcelona, Barcelona, Spain; ^3^Clinical Research Department, National Institute of Perinatology, Mexico City, Mexico

**Keywords:** cell free DNA testing, invasive testing, prenatal genetic counseling, randomized-controlled trial, fetal aneuploidy detection

## Abstract

**Objective:**

To assess the impact of prenatal genetic counseling on the attitudes and preferences toward invasive testing in first-trimester pregnant women.

**Methods:**

This is a randomized open-label study, of pregnant women undergoing first trimester combined screening for aneuploidies. Women were divided into the experimental or control groups in a 1:1 design. The intervention consisted of 15-min extra counseling about prenatal screening and diagnosis. The main outcome was the desire to choose an invasive testing as their first prenatal testing option which was measured as absolute risk.

**Results:**

After excluding those with incomplete data, 75 women remained in the experimental group and 75 as controls. Women receiving counseling were 32% more likely to choose an invasive prenatal testing as their first-line option after extra 15-min extensive counseling, reducing the first-trimester combined screening by 20% and the cell-free DNA by 12%. If given the opportunity, 59% of the women would like to be able to choose the prenatal test that suits their needs.

**Conclusion:**

Women given an extensive prenatal counseling are more likely to choose an invasive testing as their first-line test in spite of the concerning risks.

**Clinical Trial Registration:**

www.ClinicalTrials.gov, identifier NCT04119349.

## Introduction

It is well established that screening for Down syndrome should be offered in the first trimester to each pregnant woman. The most common screening method is nowadays the first trimester combined test which consists of a Bayesian analysis of the *a priori* risk of maternal age for Down’s syndrome, and the posterior risk combining serum biomarkers such as beta fraction of the human chorionic gonadotropin (β-hCG), pregnancy associated plasma protein A (PAPP-A), and nuchal translucency measurement (Nicolaides, 2003; Alldred et al., 2017). Women at high risk for trisomy 21 or 18 using this combined test are eligible for chorionic villous sampling or amniocentesis for a final diagnosis.

In recent years there has been a huge advance in prenatal screening for Down’s syndrome with the advent of cell free DNA testing with higher sensitivity and specificity than the combined test, in which a positive result must be also confirmed by an invasive diagnostic procedure. But as the range of options broadens, also the need for health education to allow women to have an adequately informed decision process on which prenatal test better suits their needs. In multicultural cities, this has become especially important to integrate patient’s values and expectations to an evidence-based decision regarding prenatal testing (Hunink et al., 2001). There is high-quality evidence demonstrating that aversion to risk of fetal loss related to an invasive test may come from incomplete information, shaping the attitude toward which test to choose from the mother’s point of view (Seror et al., 2019). And the disbelief that by taking cfDNA testing the risk of miscarriage would be reduced (Wulff et al., 2016; Malan et al., 2018).

Many information is available about preferences and attitudes in prenatal testing from Northern European studies, but scarce information is available from Southern Europe, where the amniocentesis rate in the firsts year of this century was as high as 40% of the urban pregnant population (Barcelona, 2006). We hypothesize that when enough information is given before the initial screening, women will overcome aversion to invasive testing and will be more likely to choose this method as their first choice when compared to women having routine care.

## Methods

### Study Design

This randomized open-label study, evaluated the impact of an extra 15-min prenatal counseling (extensive counseling) before undergoing first trimester combined test on the women’s attitudes and preferences toward invasive tests compared to those without extra counseling. The study protocol was approved by the local ethics committee as part of an ongoing cohort recruiting first trimester pregnancies during their antenatal assessment (HCB/2019/0788). The data were collected by the study-site manager and stored in an electronic data-capture database. The coordinator of the study along with the statistician analyzed the data and witnessed its accuracy. All authors contributed to the interpretation of the results and the preparation, approval and review of the manuscript. No private sponsor contributed to the planning, design, or conduct of the study. Protocol and study design was prospectively published on ClinicalTrials.gov under the ID: NCT04119349. We adhere to the CONSORT guidelines for clinical trials.

### Study Site

The study was conducted at Hospital Maternitat of Barcelona, a third level obstetrics and neonatal reference hospital that concentrates all first trimester screening for aneuploidies in the public health system of Barcelona. Even though this is a reference hospital for maternal-fetal pathology, all screenings for aneuploidies are performed at this site, representing the overall population (high-risk and low-risk for chromosomal abnormalities).

### Participants

Inclusion criteria were any pregnant woman attending the first trimester combined screening test for chromosomal abnormalities (between 11 + 0 and 13 + 6 weeks’ gestation), before the initial scan was performed or any combined risk calculated. The exclusion criteria were women not willing to participate in the study, or women with no sufficient knowledge of Spanish or Catalan to be able to read and understand the questionnaire.

### Intervention and Procedures

Women attending this hospital for first trimester screening for aneuploidies are gathered in the same waiting room to perform the first trimester ultrasound. As women arrived at the waiting room a clinical nurse approached each patient consecutively and asked them to participate in the study explaining the rationale of the research. Women deciding to participate were taken into an empty office where they were randomized by the study manager. Those allocated to the experimental group were taken into a second office were a maternal-fetal medicine specialist delivered the intervention, while those allocated to the control group were immediately given the survey about knowledge, attitudes and preferences of prenatal testing.

In the experimental group, participants were given extensive prenatal counseling before their first trimester scan. The extra counseling, which was the intervention in the experimental group, consisted of an explanation of all screening techniques for chromosomal abnormalities, including the first trimester combined test, cfDNA testing, invasive testing, and no screening at all. Annex 1 in the Supplementary Material shows the standardized information sheets used for counseling. The maternal-fetal medicine specialist explained pros and cons of all methods and gave time to solve any doubt the woman had. After the intervention (counseling), all participants were asked to fill a questionnaire of 21 question regarding their knowledge, attitude and preferences about prenatal testing.

In the control group, women were not given extensive counseling (no intervention), but were directly asked to fill the questionnaire about their knowledge, attitude, and preferences about prenatal testing.

### Sample Size

Sample size calculation was made using an independent two-sample proportions likelihood-ratio test. We expected a 10% of women willing to have an invasive procedure as a first line test for aneuploidies in the control group if they were given the opportunity to choose which prenatal test they would prefer, and a 25% expected difference to those in the experimental group that were given an extensive counseling, yielding a sample of 42 women per group with a Type I error of 0.0501 and a power of 80% using a two-sided test. We decided to include 80 women per arm in despite the power calculation to avoid underpowered groups due to the exclusion of participants.

### Randomization and Intervention

Participants were randomized using an interactive Web-response system assigning patients in a 1:1 ratio to receive the extra counseling before first trimester assessment (intervention group) or nothing (control group). The allocation was performed by the study manager. A clinical nurse was in charge of the participant’s enrollment and a fetal medicine specialist performed the intervention to the corresponding participants. During the study, specialists and women were aware of the allocation and intervention every time.

### Follow-up After the Intervention

After both groups (the experimental and control) filled the given questionnaire, the routine prenatal care was given according to our hospital’s guidelines, which consists of the first trimester ultrasound scan, subsequent risk calculation for trisomy 21 and 18 with the use of the first trimester combined test, and prenatal counseling. In case of risk greater than 1/250, an invasive diagnostic test was offered. For risks between 1/250 and 1/1000 a first trimester genetic sonogram by secondary markers (nasal bone, tricuspid regurgitation, and ductus venosus) was offered to re-calculate the risk for Down’s syndrome (Illa et al., 2013). For women with Down’s risk greater than 1/1000, no further evaluations were offered.

### Questionnaire

The questionnaire consisted of 21 questions assessing the knowledge, preferences and attitude toward prenatal testing in the first trimester of pregnancy. Before applying the questionnaire to the patients, we performed a pilot test with 20 patients to ensure that questions and responses were clear. The questionnaire was divided in two parts. The first 14 questions consisted of demographic characteristics and obstetrics history, such as age, ethnicity, study level, marital status, religion, salary, employment, parity, previous miscarriages, previous terminations of pregnancy, previous congenital defects, pregnancy search time in months, type of conception, and who had provided any type of previous information about prenatal screening/testing. Women were asked to select only one option for each question. All questions had the possibility to answer as “Prefer not to answer.” The second part of the questionnaire assessed the preferences and attitudes toward prenatal testing in which women were also asked to choose only one option for each question. The following questions were included in the second part of the questionnaire: What influences you the most about prenatal testing? Who influenced you the most about prenatal testing? Would you like to choose your prenatal test? Which prenatal test would you choose? What is your opinion about termination? All questions included an “I do not know answer” to also reflect the indecision that women may have about prenatal testing. There were two final questions asking, “What information regarding the results of prenatal testing is more important for you?” and “What is most important for you about prenatal testing?” Women were asked to score the first one from 1 to 5, meaning 1 the least important and 5 the most important for the first question, and from 1 to 6 in the same manner for the second question. These two questions were assessed as means, where the question with the highest mean represents the most important for the patient and the one with the lowest the least important. Annex 2 of the Supplementary Material shows the questionnaire.

### Outcomes

The primary outcome of the study was the desire to choose an invasive diagnostic testing as the first option of screening for chromosomal abnormalities. This was measured in the questionnaire by asking the question: Which prenatal test would you choose if given the opportunity? Women were asked to choose only one answer between first trimester combined test, cell-free DNA, invasive testing.

### Statistical Analysis

Analysis were conducted by intention-to-treat. Missing data for the main outcome was handled by deletion of the whole participant. All analyses were divided by groups (control vs. experimental group). Continuous data was assessed for normality using the Kolmogorov–Smirnoff test. Normally distributed variables were compared using *t*-test and expressed as mean and standard deviation (SD), while not normally distributed variables were analyzed using the Mann–Whitney U test and expressed as medians and interquartile range (IQR). Quantitative variables were compared using χ^2^ test and expressed as numbers (*n*) and proportions (%). Preferences for prenatal testing among groups were analyzed using absolute risk increase defined as the incidence of the outcome in the experimental group minus the incidence in the control group. Results for the absolute risk increase were depicted in a forest plot. Also, a multivariate logistic regression was performed to determine the odds for choosing an invasive testing adjusting by demographic characteristics and previous counseling. Data was analyzed using STATA v.15.3 for Mac (Texas College Station) and GraphPad Prism version 8.1.2 for Mac, GraphPad Software, San Diego, CA, United States, www.graphpad.com.

## Results

### Participants and Recruitment

**FIGURE 1:**
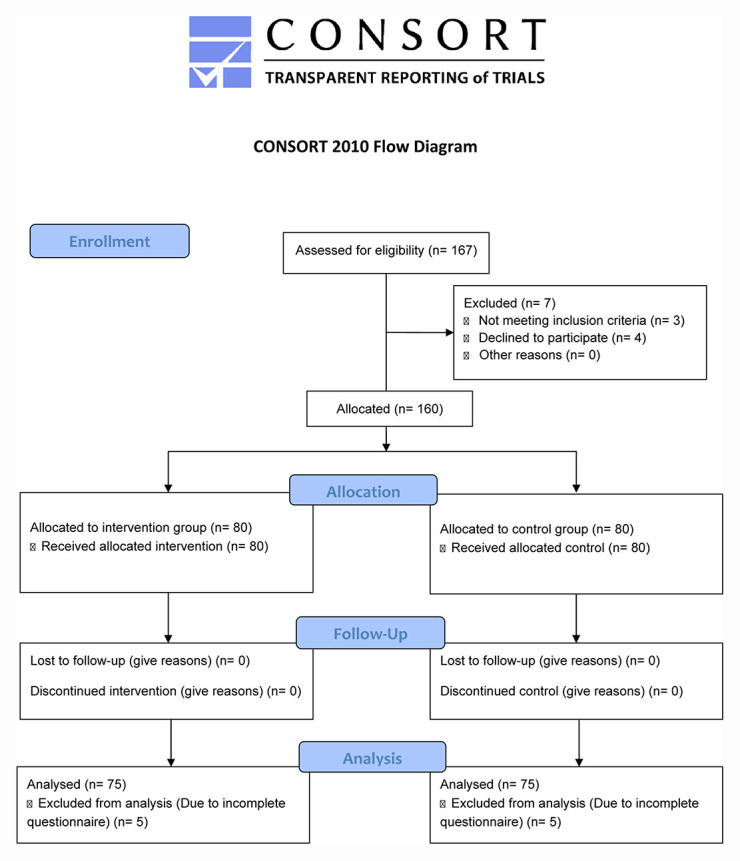
CONSORT flow diagram for transparent reporting of trials showing the number of women eligible, those allocated, followed up, analyzed and reasons for exclusion.

A total of 175 patients were eligible for allocation. After eligibility, seven pregnant women were excluded because gestation was beyond 13 + 6 weeks (*n* = 3), because they declined to participate in the study (*n* = 4), or due to an insufficient knowledge on Spanish or Catalan (*n* = 8), leaving a total of 160 women for allocation. The experimental group consisted of 80 women which were given the intervention and the control group of 80 women with no intervention. Finally, five women were excluded from each group due to incomplete data in the question related to the main outcome, leaving participants 75 for analysis in each group. The recruitment period started on October 10th, 2019. Sessions were give twice a week for an average of 21 patients per session, recruitment ended in November 11th, 2019. Figure 1 shows the CONSORT flow diagram for included and excluded participants.

### Baseline Data

The median age at inclusion was 34 (IQR: 7) years old, with no significant differences between the two groups. Fifty-five percent (*n* = 82) of the included population were white Europeans, followed by a Latin-American population of 30% (*n* = 45), Asians 9% (*n* = 13), and other ethnic groups represented the 6% (*n* = 9) of the explored population. Only one person chose not to answer this question. There were no differences in baseline characteristics among groups. Baseline characteristics are depicted in Table 1.

**TABLE 1:** Characteristics of the included population.

	Total (*N* = 150)	Controls (*n* = 75)	Informed (*n* = 75)	
*Characteristic*				
Age, median (IQR)	34 (7)	34 (8)	33 (6)	0.188
**Ethnicity, *n* (%)**				0.089
White European	82 (55)	39 (52)	43 (57)	
Latin-American	45 (30)	24 (32)	21 (28)	
Asian	13 (9)	6 (8)	3 (4)	
Pakistani/Indian	4 (3)	4 (5)	0	
Other	9 (6)	2 (3)	7 (9)	
Preferred not to answer	1 (1)	0	1 (1)	
**Study level, *n* (%)**				0.613
Primary school	2 (1)	2 (3)	0	
Secondary school	32 (21)	16 (21)	16 (21)	
Technical institute 1	17 (11)	6 (8)	11 (15)	
Technical institute 2	23 (15)	13 (17)	10 (13)	
University	71 (47)	35 (47)	36 (48)	
Preferred not to answer	5 (3)	3 (4)	2 (3)	
**Marital status, *n* (%)**				
Single	13 (9)	7 (9)	6 (8)	0.916
Married/partnership	125 (83)	61 (81)	64 (85)	
Others	8 (5)	5 (7)	3 (4)	
Preferred not to answer	4 (3)	2 (3)	2 (3)	
**Religious, *n* (%)**	58 (39)	35 (47)	23 (31)	0.091
Preferred not to answer	3 (2)	1 (1)	2 (3)	
**Salary, *n* (%)**				1
<750€	25 (17)	12 (16)	13 (17)	
751–1500€	45 (30)	23 (31)	22 (29)	
1501–2250€	30 (20)	15 (20)	15 (20)	
>2251€	34 (23)	17 (23)	17 (23)	
Preferred not to answer	16 (11)	8 (11)	8 (11)	
**Employed, *n* (%)**	113 (75)	60 (80)	53 (71)	0.404
Preferred not to answer	2 (1)	1 (1)	1 (1)	
**Parous, *n* (%)**	67 (45)	34 (45)	33 (44)	0.870
Preferred not to answer	0	0	0	
**Any previous abortions, *n* (%)**	47 (31)	24 (32)	23 (31)	0.860
Preferred not to answer	0	0	0	
**Voluntary abortions, *n* (%)**	24 (18)	9 (14)	15 (20)	0.358
Preferred not to answer	13 (9)	12 (16)	1 (1)	
**Previous congenital defects, *n* (%)**				0.173
Previous child	7 (5)	5 (7)	2 (3)	
Relatives	8 (5)	2 (3)	6 (8)	
None	113 (75)	54 (72)	59 (79)	
Preferred not to answer	22 (15)	14 (19)	8 (11)	
**Time search for pregnancy (months), median (IQR)**	4 (11)	6 (22)	3 (5)	0.066
**Type of conception, *n* (%)**				0.401
Natural	131 (87)	62 (83)	69 (92)	
Artificial insemination	2 (1)	1 (1)	1 (1)	
*In vitro* fertilization	9 (6)	7 (9)	2 (3)	
Egg donation	5 (3)	3 (4)	2 (3)	
Preferred not to answer	3 (2)	2 (3)	1 (1)	
**Who provided previous information about prenatal testing? *n* (%)**				0.872
Doctors	32 (21)	18 (24)	14 (19)	
Midwife	67 (45)	30 (40)	37 (49)	
Internet	8 (5)	4 (5)	4 (5)	
Friends	5 (3)	3 (4)	2 (3)	
Others	3 (2)	2 (3)	1 (1)	
No answer	35 (23)	18 (24)	17 (23)	

*SD, standard deviation; IQR, interquartile range (p75-p25); n, number.*

### Primary Outcome

**FIGURE 2:**
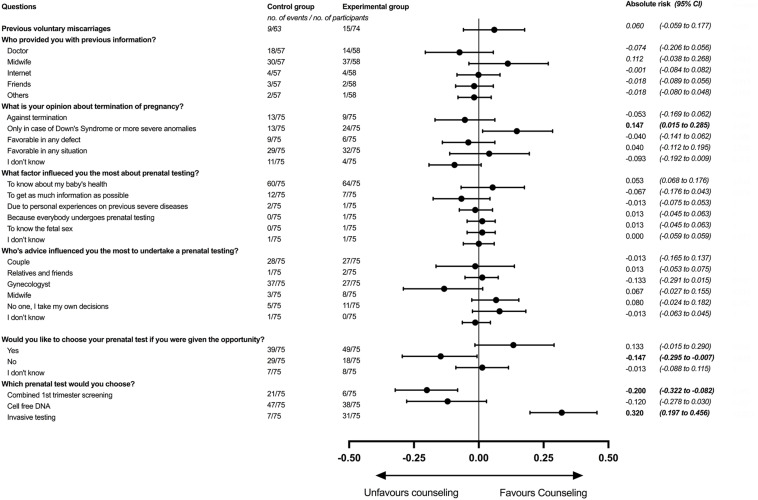
Forest plot on the calculate absolute risk for each answer in the questionnaire comparing those women allocated in the intervention and control group. Numbers on the **right side** of the graph represent the absolute risk favoring counseling, while those answers on the **left side** favor no counseling.

The primary outcome was the willing for the woman to have an invasive testing as first line option, before knowing her actual risk. Overall, 25% (*n* = 38) of women undertake an invasive test as their first choice (Table 2). In women allocated to no previous counseling, only 9% (*n* = 7) would have an invasive testing if they were given the opportunity to choose. For those given an extensive counseling, 41% (*n* = 31) would be willing to have an invasive testing as a first choice, meaning that giving extra counseling would result in a 32% risk increase (19.7–45.6%; *p* < 0.001) for choosing an invasive test as their first option. Also, if given the opportunity, women in the experimental group were less likely to choose the combined 1st trimester screening as their first choice if given (ARI: −20%; 95% CI: −32.2 to −8.2%; *p* = 0.003), while cfDNA would have a 12% reduction (−27.8 to 3%; *p* = 0.187). Figure 2 shows the forest plot for all absolute risk calculations along with their 95% CI and *p*-values.

**TABLE 2:** Questions about the knowledge and preferences regarding prenatal testing.

Questions	Total (*N* = 150)	Controls (*n* = 75)	Informed (*n* = 75)	*p*-value*
**What is your opinion about termination of pregnancy?**				
Against termination	22 (15)	13 (17)	9 (12)	0.063
In case of Down’s syndrome or more severe anomalies	37 (25)	13 (17)	24 (32)	
Favorable in any defect	15 (10)	9 (12)	6 (8)	
Favorable in any situation	61 (41)	29 (39)	32 (43)	
I do not know	15 (10)	11 (15)	4 (5)	
**What influences you the most about prenatal testing?**				
Worried about baby’s health	124 (83)	60 (80)	64 (85)	**0.030**
To know as much as possible	19 (13)	12 (16)	7 (9)	
Personal experience on severe diseases	3 (2)	2 (3)	1 (1)	
Is done by many people	1 (1)	0	1 (1)	
Knowing the fetal sex	1 (1)	0	1 (1)	
I do not know	2 (1)	1 (1)	1 (1)	
**Who influenced me about prenatal testing**				
Couple	55 (37)	28 (37)	27 (36)	0.288
Relatives and friends	3 (2)	1 (1)	2 (3)	
Gynecologist	64 (43)	37 (49)	27 (36)	
Midwife	11 (7)	3 (4)	8 (11)	
I take my own decisions	16 (11)	5 (7)	11 (15)	
I do not know	1 (1)	1 (1)	0	
**What information regarding the results of prenatal testing is most important for you? Mean (SD)**				
To know the fetal sex	1.32 (1.07)	1.14 (0.52)	1.49 (1.40)	0.055
Down syndrome	**2.97 (1.18)**	**2.75 (1.02)**	**3.18 (1.29)**	**0.028**
Major chromosomal abnormalities	4.04 (0.95)	3.9 (0.94)	4.17 (0.95)	0.084
Minor chromosomal abnormalities	**4.09 (1.13)**	**4.29 (0.94)**	**3.89 (1.13)**	**0.021**
Any cause of mental disability	3.57 (1.18)	3.54 (1.17)	3.6 (1.19)	0.760
**What is most important for you about prenatal testing? Mean (SD)**				
Early in pregnancy	**3.59 (1.60)**	**3.26 (1.62)**	**3.89 (1.53)**	**0.016**
Short waiting list	3.15 (1.38)	3.19 (1.47)	3.11 (1.30)	0.723
Know more fetal anomalies	5.41 (1.11)	5.28 (1.20)	5.53 (1.02)	0.172
At no cost	2.78 (1.64)	2.65 (1.60)	2.89 (1.69)	0.381
Less risk of abortion	4.6 (1.38)	4.66 (1.38)	4.55 (1.39)	0.632
Less annoyance for pregnancy	2.57 (1.55)	2.54 (1.43)	2.59 (1.66)	0.859
**Would you like to choose your prenatal test?**				
Yes	88 (59)	39 (52)	49 (65)	0.157
No	47 (31)	29 (39)	18 (24)	
I do not know	15 (10)	7 (9)	8 (11)	
**Which prenatal test would you choose?**				
First trimester combined test	27 (18)	21 (28)	6 (8)	<0.001
cfDNA testing	85 (57)	47 (63)	38 (51)	
Invasive test	38 (25)	7 (9)	31 (41)	

*Bold values are “statistical significant”.*

### Preferences About Prenatal Testing and Opinions About Abortion

Women receiving extra counseling had 14.7% (1.5 to 28.5%; *p* = 0.058) higher risk to say that abortion should be given only in case of Down’s syndrome. When asked about factors influencing them the most about prenatal testing 83% answered that their main objective was to know about their baby’s health, though no significant differences were found among the other answers. The gynecologist (43%) and the couple (37%) where the ones influencing the most in the decision to undergo prenatal testing, while the majority of the participants (59%) say that they would like to be able to choose their prenatal testing if given the opportunity, especially after having an extra counseling (13.3%; −1.5 to 29%; *p* = 0.136).

When women were asked to score from the least important to the most important what is most important for them about prenatal testing and what is it that they feel is more relevant; the highest importance was given to “know more fetal anomalies,” “know minor chromosomal anomalies,” and “major chromosomal anomalies.” Nonetheless, when comparing both studies groups, significant differences were found about the importance of Down’s syndrome, being of higher importance among counseled women, and minor chromosomal abnormalities being more important among controls. Regarding the relevance about prenatal testing, women in the experimental groups felt that performing prenatal testing early in pregnancy was more important when compared to the controls (Table 2).

### Factors Influencing the Willing to Choose an Invasive Prenatal Testing

**FIGURE 3:**
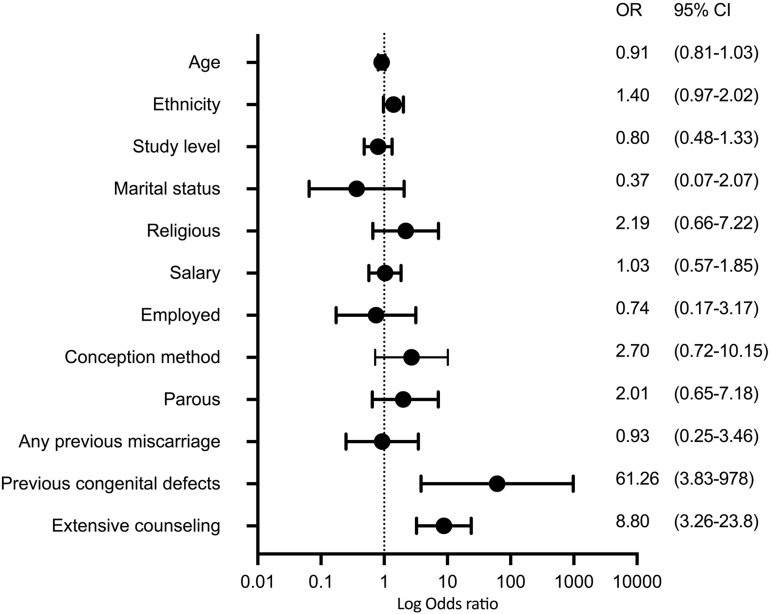
Forest plot showing the odds ratio for the association between the willing to have an invasive procedure as first-line prenatal testing to the demographic characteristics of the included population after adjusting to previous counseling and all variables between each other.

A multivariate logistic regression was performed to assess the influencing factors behind the willing to choose an invasive procedure as a first line test by adjusting to the baseline characteristics. Women with history of previous child with congenital defects had higher odds for choosing an invasive testing (aOR: 61.3; 3.83–0978; *p* = 0.004), while having an extensive counseling was significantly associated with the willing to undergo an invasive testing (aOR: 43.9; 5.78–332; *p* = < 0.001). Figure 3 shows the forest plot with their respective log-scaled odds ratios.

## Discussion

### Main Findings

In this experimental study involving pregnant women undergoing first trimester scan and aneuploidy screening, when given extensive counseling prior to the screening were 32% more likely to choose an invasive prenatal testing as their first option and 20% less likely to choose the first trimester combined test, as compared to those without counseling. Also, if given the opportunity, 59% of the women would like to choose their own prenatal testing, which was 13.3% higher in those given extra counseling, significantly reducing the “no” as an answer (14.7%).

In our study comprising a multicultural population from very different ethnic backgrounds and study levels, 77% of the women had previous information on prenatal testing, provided mainly by midwifes and also doctors. Most of our pregnant population would undergo prenatal testing because of worry about their baby’s health. Nevertheless, when asked who influenced them the most for taking a prenatal test, the doctor’s opinion was the strongest, followed by the opinion of their couple. The influence of prenatal counseling toward reducing the uncertainty in pregnant woman was remarkable. Those receiving counseling were less likely to answer, “I do not know”, and more likely to answer “yes” when asked if they would like to choose their prenatal test if given the opportunity.

### Comparison With the Literature

Most of the studies on women attitudes and preferences in relation to prenatal testing were carried out in Northern Europe, particularly Denmark and Netherlands. In Denmark, the country where first trimester combined test was best organized, 99.6% of women chose to have risk assessment with a high degree of knowledge (82%) and positive attitudes regarding risk assessment (97%), leading to 79% making an informed choice. At 30 weeks of pregnancy, 99% of women were satisfied with having chosen risk assessment (Bangsgaard and Tabor, 2013). Another Danish study, this in a high-risk population demonstrated that 75% of women chose an invasive test and 24% chose cfDNA testing. Choosing cfDNA was found to be associated with a high decisional conflict and therefore lower satisfaction with the genetic counseling was associated with low decisional conflict and later decisional regret (Hartwig et al., 2019). In Sweden, a divided attitude toward invasive testing was found in high school students because 29% of them showed uncertainty about this method, while 48% had a very positive attitude toward it (Georgsson et al., 2017). In Norway, a majority of women supported increased access to prenatal screening with ultrasound (60%) and/or full genome sequencing of fetal DNA (55%) available for all pregnant women, although significant minorities indicate, however, that a public offer of prenatal screening for all pregnant women would signal that people with Down syndrome are unwanted or could be criticized for contributing to a “sorting society” (Magelssen et al., 2018). In Netherlands, where conventional Down syndrome screening had a low uptake (46%), a study showed that the introduction of cfDNA testing might allow couples to decide about prenatal testing based mostly on their will to test or not, rather than largely based on fear of miscarriage risk or the uncertainty of results (Van Schendel et al., 2014). In another Dutch study, interviews performed in clinicians and pregnant women showed significant differences in cfDNA/invasive testing ratios after a high-risk result between women and clinicians, with the largest difference being 35 vs. 4% opting for invasive testing (Van Der Steen et al., 2019). In an international study comparing the preferences on prenatal testing between women and health professionals, demonstrate that women assign a relatively higher value to test safety and having comprehensive information, while health professionals place more emphasis on accuracy and early testing than women do. Women prefer a test with no risk of miscarriage as they were prepared to wait more than twice as long and accept 6 percentage points lower accuracy compared with health professionals for a test that had no miscarriage risk. Furthermore, women were prepared to wait more than twice as long and accept a 2-percentage point decrease in accuracy compared with health professionals for a test that gave comprehensive information (Hill et al., 2016). In the same study, when preferences are compared between the participating countries (Canada, Denmark, Iceland, Israel, Italy, Netherlands, Portugal, Singapore, and the United Kingdom) shown that Italian and Portuguese women were those placing lower emphasis on safety, although the old 1% rate was still used, in the opposite extreme than Dutch and British women. In Southern Europe, women prefer invasive testing than cfDNA and they are more prepared to accept test with a miscarriage risk to gain more comprehensive information than women from other countries (Hill et al., 2016). This is not completely unexpected since in the nineties the amniocentesis rate approached 40% of the urban pregnant population in the cities of France, Spain, and Italy.

In our study population, a 25% average preference for invasive testing does not reflects the real situation since only 9% of women without counseling would choose an invasive test, but after counseling this rate would increase to 41%, accounting for a 32 percentage points increase. This remarkable change on the attitude toward invasive testing reflects a lack of knowledge. When sufficient information is received, women would clearly prefer an invasive testing rather than the combined test, reducing the combined 1st trimester screening and cfDNA as their first line option. Other investigators have found that the knowledge and attitude toward prenatal testing change in different countries of the world. A study from Egypt (Eldin et al., 2017) found that 53% of the 351 studied women did not know about the availability and accuracy of prenatal screening tests, but after counseling 78% of the women had a positive attitude toward prenatal screening. Similarly, In Rumania, Pop-Tudose et al. (2018), found that 48% of women have never have heard about any test for Down’s syndrome detection. Differently to this Abdo et al. (2018), found that more than 94% of Jordanian women would consider having a prenatal testing, specially cfDNA testing, although this rate was lower in women with less education.

### Translation to the Clinical Setting

Our results imply that changes in the implementation of prenatal testing should be accompanied by previous extra counseling and health education to reduce uncertainty among the population and to help women to choose which is the prenatal test that suits better their needs and preferences. Information could be handled by pamphlets (Marteau et al., 1993; Glazier et al., 1997; Nagle et al., 2008), counseling (Thornton et al., 1995; Hewison et al., 2001) or audiovisuals (Graham et al., 2000; Leung et al., 2004; Kuppermann et al., 2009), which have demonstrated high satisfaction among woman. The possibility of choosing their own method for prenatal testing sits at the peak of women’s empowerment by adapting the patient’s values and preferences to the best available evidence, a step at which we must aim as society.

### Strengths and Limitations

The strength is this article is the experimental design and the rigorous methodology to implement an adequate analysis according to the best possible standards, reassuring the validity of the results. For limitations, it could be argued that the fetal-medicine specialist giving the extensive counseling could be biased because of the awareness of the allocation for each patient. Nevertheless, this reflects the every-day interaction that the personnel involved in prenatal counseling have with their patients on a human level.

## Conclusion

Women receiving prenatal counseling are more likely to decide that they want to choose an invasive testing over the combined 1st trimester screening. This shows the impact that health education has on informed decision and the importance of empowering women by adapting the best possible evidence to their values and preferences from an evidence-based point of view.

## Data Availability Statement

The raw data supporting the conclusions of this article will be made available by the authors, without undue reservation.

## Ethics Statement

The studies involving human participants were reviewed and approved by Hospital Clinic Ethics Committee under approved protocol (HCV/2019/0788). The patient/participant provided their written informed consent to participate in the study.

## Author Contributions

FP contributed to the design and implementation of the research, to the analysis of the results, and to the writing of the manuscript. RM-P and MP analyzed the data. AB supervised the project and conceived the original idea. All authors discussed the results and contributed to the final manuscript.

## Conflict of Interest

The authors declare that the research was conducted in the absence of any commercial or financial relationships that could be construed as a potential conflict of interest.
