# Supplementary material for: A Randomized Controlled Trial on the Influence of Prenatal Counseling on the Attitudes and Preferences Toward Invasive Prenatal Testing Among Women in Their First Trimester of Pregnancy (INVASIVE)
Source: Front Genet. 2020 Nov 9;11:561283. doi: 10.3389/fgene.2020.561283 (PMC7682740; doi:10.3389/fgene.2020.561283)
Supplement: Supplementary file 1 [file Presentation_1.PPTX]

## Slide 1
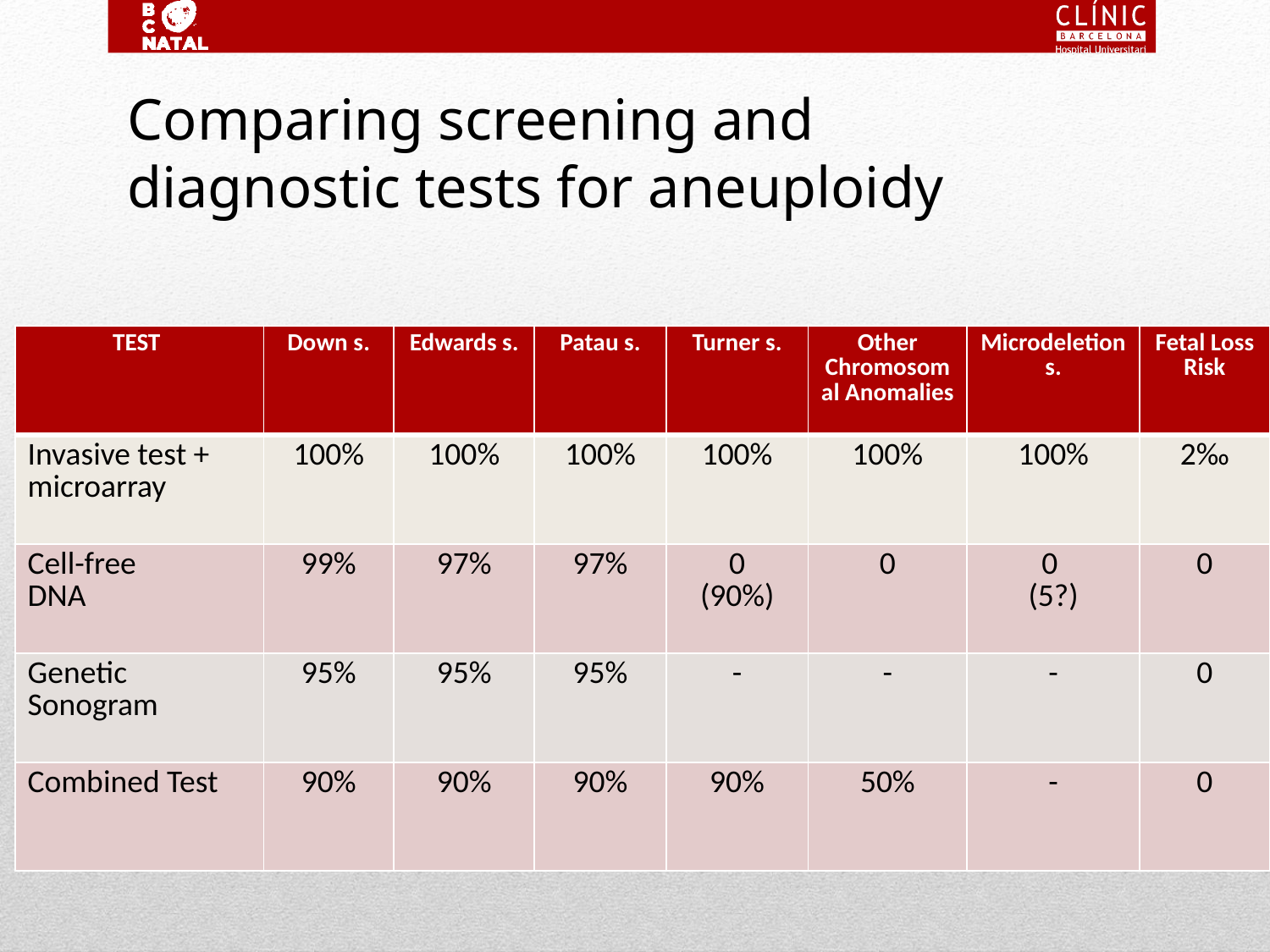

Comparing screening and diagnostic tests for aneuploidy
| TEST | Down s. | Edwards s. | Patau s. | Turner s. | Other Chromosomal Anomalies | Microdeletion s. | Fetal Loss Risk |
| --- | --- | --- | --- | --- | --- | --- | --- |
| Invasive test + microarray | 100% | 100% | 100% | 100% | 100% | 100% | 2‰ |
| Cell-free  DNA | 99% | 97% | 97% | 0 (90%) | 0 | 0 (5?) | 0 |
| Genetic Sonogram | 95% | 95% | 95% | - | - | - | 0 |
| Combined Test | 90% | 90% | 90% | 90% | 50% | - | 0 |

## Slide 2
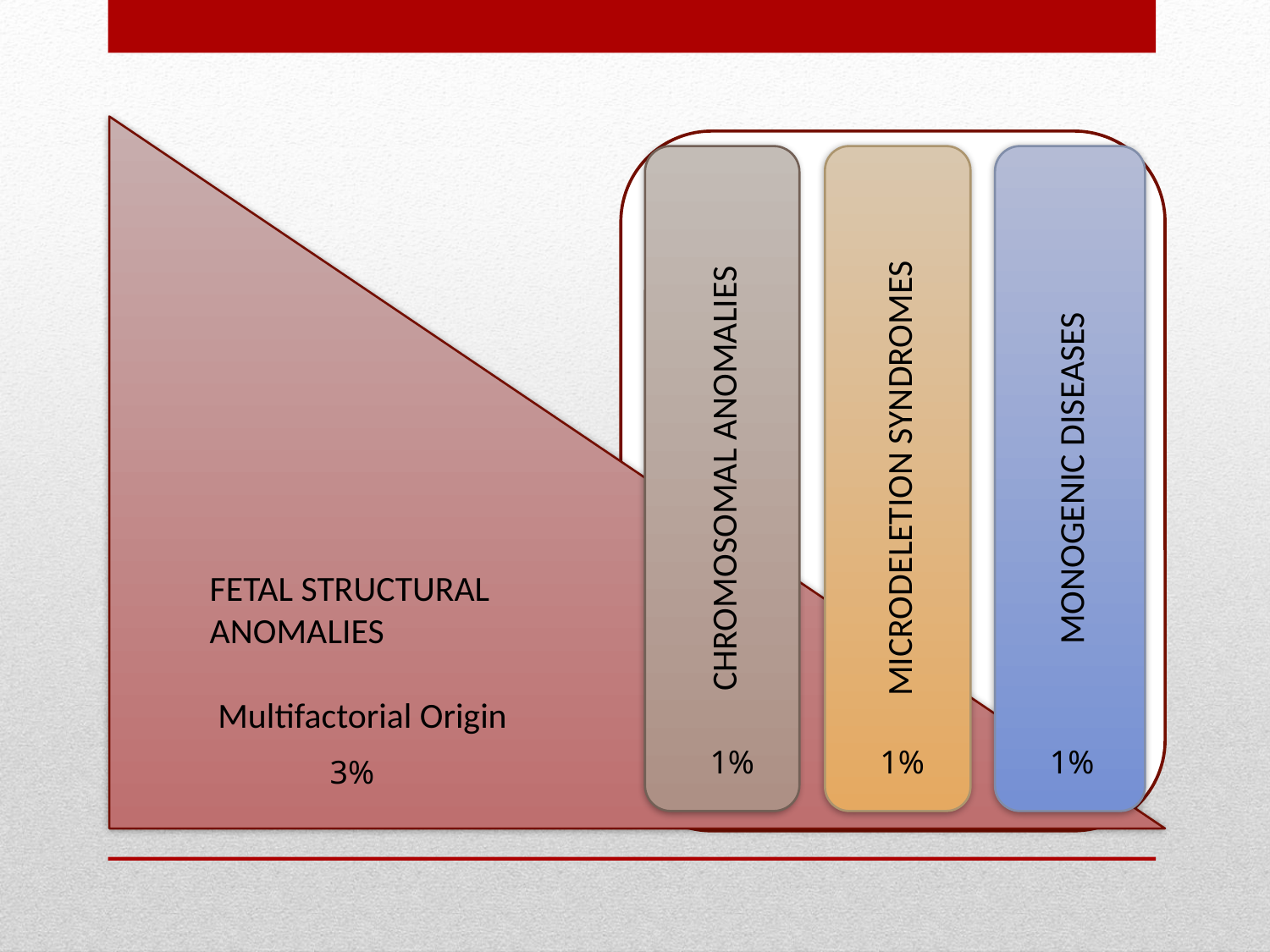

FETAL STRUCTURAL
ANOMALIES
 Multifactorial Origin
CHROMOSOMAL ANOMALIES
MICRODELETION SYNDROMES
MONOGENIC DISEASES
1%
1%
1%
3%
