# Supplementary material for: A Randomized Controlled Trial on the Influence of Prenatal Counseling on the Attitudes and Preferences Toward Invasive Prenatal Testing Among Women in Their First Trimester of Pregnancy (INVASIVE)
Source: Front Genet. 2020 Nov 9;11:561283. doi: 10.3389/fgene.2020.561283 (PMC7682740; doi:10.3389/fgene.2020.561283)
Supplement: Supplementary file 2 [file Table_1.DOCX]

**Knowledge, Attitudes, and preferences regarding prenatal tests for the detection of genetic abnormalities in pregnant women of BCNatal-Maternitat**

Name and surname ______________________________________________________________

Random Number_______ Phone ______________________

Date of Birth___________________

Code _________ Mail________________________________

A) SOCIO-DEMOGRAPHICAL AND OBSTETRIC CHARACTERISITCAS.

1. Age ____

2. Ethnicity:

Caucasian ____ Latin ____ Asian ____ Afro descendant ____ Other____

3. Schooling

Preschool____ Primary ____ Secondary____ Cycle Form. Medium ____

Form cycle. Superior ____ University____

4. Marital Status

Single____ Married ____ Widow ____ Separated____

5. Do you profess any religion?

Yes ____ NO____

6. Occupation

Employee ____ Unemployed / Unemployment ____

7. Net monthly income of the family unit (euros)

<750 ____ 751-1500 ____ 1501-2250 ____ > 2250____

8. Do you have previous children? NO____ YES ____ How many? ____

9. Have you had previous miscarriages? NO____ YES ____ How many? ____

10. Have you had previous voluntary abortions? NO____ YES ____ How many? ____

11. Congenital defects in: previous children ____ relatives____ there is no history ____

12. Pregnancy search time: ____

13. Conception mode

Natural ____

Assisted ____ a. Ovarian stimulation ____ b. Artificial insemination ____

                           c. In- vitro Fertilization (IVF) ____ d. IVF with donor eggs ____

**B) INFORMATION ABOUT PRENATAL TESTS**

14. Have you received information about prenatal diagnostic tests?

Yes____ No____

15. Who did you receive the information on prenatal tests from?

Doctor ____ Midwife____ Internet ____ Friends ____ Others ____

INFORMATION ABOUT PRENATAL TESTS

16. What information from prenatal tests do you consider to be the most important?

Order from more (1) to less (5) important:

- Fetal sex ____

- If the fetus has Down syndrome ____

- If the fetus has a more serious chromosomal abnormality than Down Syndrome____

- Any detectable chromosomal abnormality is more or less severe ____

- Any cause of mental retardation ____

17. What is most important to you about prenatal testing?

        Put a number from 1 to 6, from most to least important:

- to be done early in pregnancy ____

- the waiting time for the results is short ____

 - diagnose the greatest amount of genetic abnormalities ____

- no economic costs for pregnant women ____

- lowest possible risk of abortion ____

- minor discomfort for the pregnant woman ____

18. What opinions do you have about voluntary abortion?

- I am against any situation ____

- I am favorable in cases like Down S. or more serious ____

- I am favorable for any defect ____

- I am in any situation in favor ____

C) ATTITUDES AND PREFERENCES ON PRENATAL TESTS

19. What influences the decision about prenatal testing?

Concern for the baby's health ____

Want to know as much as possible____

There is no reason to refuse them ____

Personal experience of serious illnesses in acquaintances ___

Many people do____

Importance of knowing the sex of the baby ____

20. What opinions influence the decision about prenatal testing?

Couple ____ Family and friends____

Gynecologist ____ Midwife ____

No one, I make the decision myself____

21. Would you like to be able to choose the prenatal test that you think can give you the information you want and that is covered by social security?

No ____ Yes____

What would it be? ______________________________________

22. PREFERENCE OF A PRENATAL TEST

Option 1. ____

Screening of the I trimester (analytical at week 10 and ultrasound between 11-13 weeks)

Down S. detection rate 90%

Result a few hours after ultrasound

If it is high risk, an invasive test will be done.

Option 2. ____

Fetal DNA in maternal blood

Down S. detection 99%

If it is positive, it needs confirmation with an invasive test.

Option 3. ____

Invasive Test (Chorionic villus sampling or Amniocentesis)

Risk of spontaneous abortion of 2 per thousand cases

100% detection of Down S. , other chromosomal abnormalities and 130 rare diseases

Initial result: 24 hours. Final result 10 days
